# Pollination Ecology, Breeding System, and Conservation of *Butia lallemantii* Deble & Marchiori (Arecaceae): A Useful Dwarf Palm Tree from the Pampa

**DOI:** 10.3390/plants13111562

**Published:** 2024-06-05

**Authors:** Oscar Perdomo, Rafael Becker, Rodrigo Bustos Singer

**Affiliations:** 1 NÚCLEO—Basic Science Research Group, Faculty of Science and Engineering, Universidad de Boyacá, Tunja 150003, Colombia; 2 Laboratory of Systematics of Vascular Plants, Postgraduate Program in Botany, Federal University of Rio Grande do Sul, Porto Alegre 91509-900, RS, Brazil; beckerr92@gmail.com (R.B.); rbsinger1@yahoo.com (R.B.S.); 3 Graduate Program in Botany (PPGBOT-UFRGS), Institute of Biosciences, Federal University of Rio Grande do Sul, Porto Alegre 91501-970, RS, Brazil

**Keywords:** *Butia*, entomophily, geitonogamy, Halictinae, Meliponinae, native pollinators, self-compatibility

## Abstract

The Dwarf Palm, *Butia lallemantii* Deble & Marchiori, is an endangered species endemic to the Pampa biome and typically grows in sandy and rocky soils. Given its economic, ecological, and cultural relevance, it is crucial to understand the ecology and biology of this species to encourage its preservation and highlight its significance for the Pampa. This study aims to investigate whether this palm relies on animal vectors for pollination, analyze its breeding system, and propose strategies for its conservation and sustainable use. We conducted field observations on pollination ecology, identified floral visitors, and designed six breeding system experiments to test cross-compatibility, self-compatibility, and apomixis. Additionally, we conducted a literature review to propose conservation strategies. *Butia lallemantii* is pollinator-dependent and self-compatible. The flowers are mostly melittophilous and offer pollen and nectar for floral visitors. The main pollinators are native Meliponinae and Halictinae bees and the introduced *Apis mellifera*. This study represents the first comprehensive and complete examination of the breeding system and pollination process on *Butia* palms. This palm can provide materials for industries, but urgent actions are needed to preserve the remaining populations through effective policies and strategies. Furthermore, this palm should be integrated into diversified agroecosystems to evaluate its adaptability to cultivation.

## 1. Introduction

The Pampa biome is a mainly grassy ecosystem located in southern South America, covering more than 700,000 km^2^ of central and Northeastern Argentina, Southern Brazil, Southeastern Paraguay, and Uruguay [1]. The landscape is dominated by natural grasslands formed by C3 and C4 grasses, with forested areas restricted to the banks of rivers and regions with more pronounced topography [2,3]. The mean annual precipitation varies from 400 to 1500 mm, with mean temperatures ranging from 13 °C to 20 °C [4]. Although the Pampa is one of the most altered and threatened biomes in Brazil, only 3% of its area is under any kind of protection, and less than 0.5% is in strictly protected areas [5,6]. In this biome, the soil is susceptible to eolic and hydric erosion due to its sedimentary rock origin, which makes anthropic activities like livestock, agriculture, and forestry more harmful to the environment and biodiversity [7]. Biodiversity reaches high levels in the Pampa. In Brazil, more than 12,500 species are registered, nearly 3700 of them being vascular plants, with a record of 56 vascular plant species in 1 m^2^ [8,9].

Only ten species of Arecaceae Bercht. & J. Presl are recorded for the Pampa, six of them in the genus *Butia* (Becc.) Becc., three in *Trithrinax* Mart., and one in *Syagrus* Mart. [10,11]. Pollination in tropical Arecaceae is mainly entomophilous; nearly 80% of known species are considered to be insect-pollinated [12,13]. Many insect species are drawn to palm flowers for food, mating places, and preying; and the abundance of those insects renders them of different importance for pollination [14,15]. Arecaceae presents a variety of pollination syndromes, with cantarophily (Coleoptera), melittophily (Hemiptera), and myophily (Diptera) being the most relevant [12,16]. On the other hand, the diversity and abundance of floral visitors attracted by a single palm species make it challenging to define a pollination syndrome [17]. Despite this, some studies have demonstrated these syndromes in Arecaceae [14,15,18,19].

*Butia* is a subtropical genus of palms distributed in Argentina, Brazil, Paraguay, and Uruguay. These palms are monoecious, with lateral inflorescences, pinnate leaves, leaflets in ascending disposition, imperfect flowers, and three pores in the endocarp [20]. To date, studies coupling both pollinator observations and detailed breeding system experiments are lacking. Diverse insects have been observed in the inflorescences of *Butia paraguayensis* (Barb. Rodr.) L.H. Bailey [15] and *Butia leiospatha* (Barb. Rodr.) Becc [21]. However, these contributions did not test (through breeding system experiments) whether or not these palm species need putative insect pollinators to set fruit and viable seeds. Indeed, since the inflorescences of *Butia* spp. bear imperfect flowers, only insects regularly visiting the two flower types can be considered pollinators. In this regard, only the study on *B. paraguayensis* identified visitors who accomplish these requirements [15]. Regarding their breeding systems, *Butia capitata* (Mart.) Becc. and *Butia eriospatha* (Mart. ex Drude) Becc. were reported as self-compatible [22,23]. However, the rest of the species of the genus have not been studied so far regarding either their pollination or their reproductive biology [12].

*Butia lallemantii* Deble & Marchiori, also known as Dwarf Butiá, is a small caespitose palm native to the Pampa biome that offers raw materials for food, drinks, handicrafts, animal feeding, and industry [24,25]. Unfortunately, many of these uses have been lost with time, and this species is currently underutilized. The few remaining populations are isolated and need management for sustainable use and conservation [24]. Recently, some populations have been characterized, evaluating the growth of ramets on different substrates for their possible use in the restoration of degraded soils [26,27,28,29]. However, it is necessary to develop more studies on the biology, ecology, and ethnobotany of this species to generate adequate policies and strategies to conserve it and promote its sustainable use.

In this research, we addressed the following questions: (i) Is *B. lallemantii* pollinator-dependent? (ii) Is the species self-incompatible? (iii) Does this species offer any rewards to their floral visitors? (iv) Who are the pollinators? We hypothesized that: (i) *B. lallemantii* is pollinator-dependent. (ii) that this species is self-compatible and geitonogamous, and (iii) these species produce nectar, as in other species of the genus [30]; (iv) the primary pollinators may be native bees, insects already observed in *Butia* species co-occurring in the Brazilian Pampa.

## 2. Results

### 2.1. Inflorescence Morphology, Anthesis, and Flower Duration

The inflorescence of *B. lallemantii* is enclosed by a spathe, and the rachis is the axis where the rachillae are inserted. Pistillate flowers are proximally located in the rachilla, with the staminate ones being distally located. There is high variability in the size of the inflorescence and its structures (Table 1).

**Figure 1:**
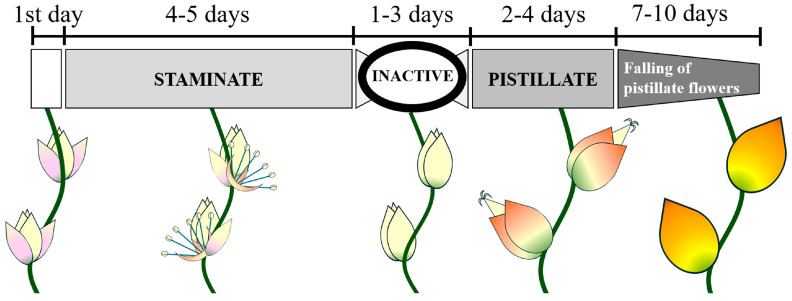
Phenophases of *Butia lallemantii* observed in the Brazilian Pampa.

The anthesis in *B. lallemantii* extends for 9 to 11 days (mean = 10.35, *n* = 20); on the first day, the spathe opens and exposes the inflorescence with unopened staminate and pistillate flowers. Thereafter, it exhibits three phenophases: the staminate phase extends for 4 or 5 days (mean = 4.55); the neutral phase without opened flowers extends for 1 to 3 days (mean = 2.15); and finally, the pistillate phase starts after 2 to 4 days from the neutral phase (mean = 2.65). Staminate flowers open in the morning, from 7 to 9 h, and last for 5 to 9 h (mean = 7.2, *n* = 25), falling between 13 and 17 h in the afternoon. The pistillate flowers opened from 7 to 10 h but stayed attached to the inflorescence for 7 to 10 days (mean = 8.52, *n* = 25) (Figure 1).

### 2.2. Vector-Dependent Pollination

After 15 days, none of the 20 inflorescences isolated to test for anemophily and pollination insect-dependence formed fruits. We did observe some pistillate flowers still attached to the rachillae of the wind-dependent experiments, but they were brown-colored, indicating tissue senescence and no fruit formation, and these flowers fell soon thereafter.

### 2.3. Self-Compatible and Geitonogamous

**Figure 2:**
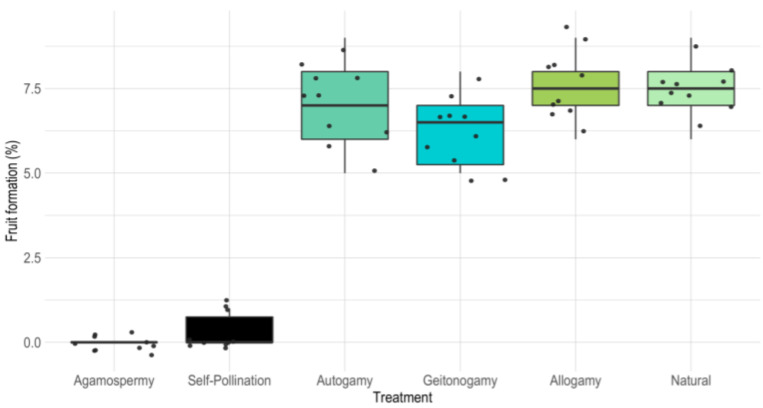
Boxplot of the results from the breeding system experiments on *Butia lallemantii,* in the Brazilian Pampa.

Our pollination tests demonstrated self-compatibility in *B. lallemantii*. Spontaneous self-pollination tests resulted in the formation of 3.3% of the fruits, whereas hand-made self-pollinations resulted in 77.8% of fruit formation and the geitonogamy tests in 70%. The natural pollination test resulted in 84.3% of fruits developing (Table 2). Hand-made cross-pollination reached 84.4% fruiting success. Only the agamospermy test resulted in 0% of fruits being formed. We compared the results of the pollination treatments using the ANOVA test, using Autogamy, Allogamy, Geitonogamy, and Natural Pollination data. Agamospermy and Spontaneous Self-Pollination were excluded from the analysis due to the non-normality of the data and the evident differences with the other treatments. The results indicate significant statistical differences between at least two groups (df = 5, F = 172.1, *p* < 0.001) (Table 3). Then, we used a pairwise Tukey test to identify the differences. Our analysis revealed that natural pollination and allogamy differed significantly from geitonogamy. Additionally, we found that hand-pollinated autogamy does not differ from hand-pollinated allogamy (Figure 2).

The Index of Self-Incompatibility value was 0.921, with 70 fruits formed by autogamy and 76 formed by allogamy, supporting the self-compatibility of the species. The reproductive efficiency was 0.574 (57.4%), with 77 fruits developed from 134 pistillate flowers.

### 2.4. Floral Resources and Pollinators

**Figure 3:**
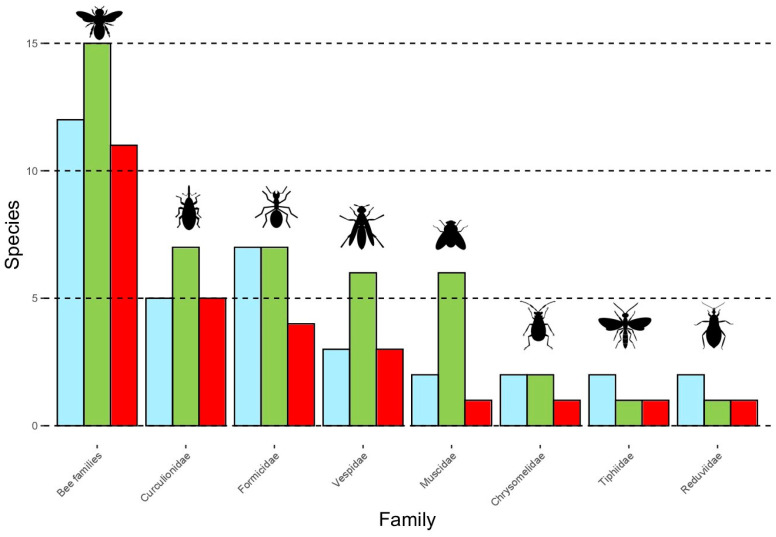
Species richness per family of floral visitors in pistillate (blue bars), staminate (green bars), and both phenophases (red bars) of *Butia lallemantii* in the Brazilian Pampa. Bee families are Apidae, Andrenidae, and Halictidae.

During our observations of the inflorescences in both the staminate and pistillate phases, we observed that floral visitors of *B. lallemantii* were actively consuming the pollen or nectar produced by the plant. We recorded 61 species of floral visitors belonging to 16 insect families and four orders (Table 4). We did not observe floral visitors interacting with the flowers at night. The most diverse groups were Bees (Apidae), ants (Formicidae), flies (Muscidae), wasps (Vespidae), and weevils (Curculionidae) (Figure 3). Bees, weevils, and flies were observed collecting and consuming nectar and pollen, while wasps and ants were observed consuming nectar and preying in both phases.

### 2.5. Bees, the Main Pollinators

The floral visitors we registered on *B. lallemantii* belong to 16 families in four orders; Hymenoptera (55.7%), Coleoptera (26.2%), Diptera (14.8%), and Hemiptera (3.3%) (Table 3) (Supplementary Material, Video S1). Some Hemiptera, Nitidulidae, Chrysomelidae, Cerambycidae, and Elateridae were present with one species, all in the staminate phase. On the other hand, Lampyridae and Syrphidae, also with one species, were observed only in pistillate inflorescences. We do not consider these families as pollinators of *B. lallemantii* because they were recorded only in one phenophase, with few individuals and low visitation rates. As the morphological features and behavior of these insects in the inflorescences may promote pollination, we do not discard this possibility.

**Figure 4:**
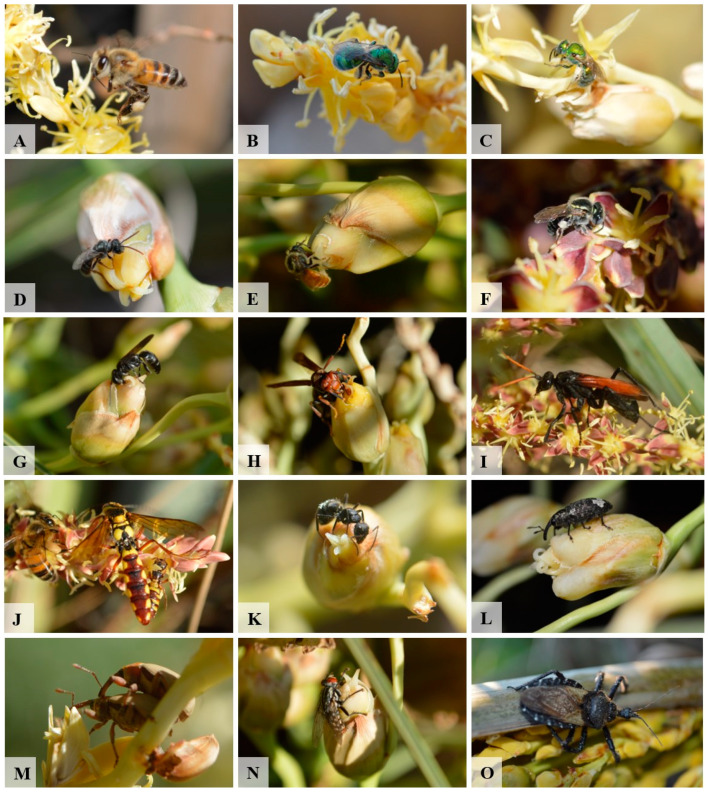
Some pollinators of *Butia lallemantii* in the Brazilian pampa. (**A**) *Apis mellifera*; (**B**) Halictidae sp1; (**C**) Halictidae sp2; (**D**) *Plebeia* sp1.; (**E**) *Plebeia* sp2; (**F**) *Plebeia* sp3; (**G**) Andrenidae sp1; (**H**) Vespidae sp1; (**I**) Vespidae sp2; (**J**) Tiphiidae sp3; (**K**) Formicidae sp1; (**L**) Molytinae sp1; (**M**) *Derelomus* sp1; (**N**) Muscidae sp1; (**O**) Reduviidae sp1.

We registered 17 bee species (Apidae, Andrenidae, and Halictidae) visiting inflorescences of *B. lallemantii*; 12 of them were observed in both staminate and pistillate phases, representing 44.4% of the pollinators identified for the species (Table 3). The bees reported in this study have morphological adaptations to carry pollen, and they actively collect and consume pollen and nectar. The bees were observed walking around the staminate flowers while collecting pollen and eventually licking nectar. In the pistillate flowers, they searched for nectar accumulation near the floral apex and passed many times over the stigma. This behavior makes them a significant group of pollinators for *B. lallemantii*. *Apis mellifera* Linnaeus (Figure 4A) was the most frequent and numerous species, registered in all the individuals observed, followed by Halictidae species (Figure 4B–D), Meliponini from the genera *Plebeia* and *Trigona* (Figure 4D–F), and an Andrenidae (Figure 4G). Small Halictidae species (Figure 4C) show a different foraging behavior, flying around the inflorescences and rachillae, posing for a few seconds (1–3), and flying again. As a whole, our results indicate bees as the primary pollinators for these palm trees, with a high contribution from the introduced honeybee.

Three of the six species of Vespidae (Figure 4H–J) we recorded were observed during both phenophases, primarily preying and consuming nectar. However, their ability to carry pollen is limited, which restricts their role in pollination and likely makes them secondary pollinators. Two species of Tiphiidae wasps were recorded consuming nectar while copulating, but only one species was recorded in both phenophases (Figure 4J). We observed ten species of Formicidae (Figure 4K), four of them visiting inflorescences in both phenophases. *Atta sexdens* was registered as collecting staminate and pistillate flowers, and they likely transfer pollen between inflorescences on their bodies. But these ants were predating the inflorescences and carrying flowers to their nests. Therefore, it is difficult to confirm their role as pollinators. Finally, we observed that their bodies load pollen while feeding, promoting pollination when visiting pistillate inflorescences.

Five of the seven species of Curculionidae (Figure 4L,M) were observed in both phenophases, and their role in pollination is indubitable, representing 18.5% of the pollinators, but their behavior is also related to predation and parasitism. Although they are recurrently reported as pollinators in Arecaceae, their role is considered secondary due to the low number of individuals and infrequent visits compared to the primary pollinators. We found only one species each of Chrysomelidae, Tiphiidae, and Muscidae (Figure 4M) in both phenophases. Therefore, we considered their role in pollination to be less significant. The unique Hemiptera was a Reduviidae species (Figure 4O) we observed hunting by stalk, mainly *A. mellifera*. Their behavior makes it improbable that they can act as pollinators.

## 3. Discussion

### 3.1. Anthesis and Floral Duration

The anthesis of *B. lallemantii* exhibits both unisexual flowers and protandrous behavior, features normally related to entomophily [21]. Staminate flowers open and fall on the same day, offering pollen and nectar for the floral visitors from the morning to the afternoon for 4–5 days. Pistillate flowers open in the morning and offer nectar throughout the day for 2–4 days. Phylogenetic studies have demonstrated an association between protandry and pollinator type, suggesting its influence on the pollination mode evolved [31]. On the other hand, protandry is not significantly correlated with biotic pollination and is considered a less effective adaptation to avoid self-pollination because the pollen may remain viable for enough time to fertilize flowers of pistillate phenophase [32,33], as we demonstrated for *B. lallemantii*.

### 3.2. Insect-Dependent Pollination

The lack of fruit formation in both the wind and insect dependence experiments supports our hypothesis that *B. lallemantii* is dependent on animal pollen vectors for pollination. Although we obtained a few fruits from spontaneous autogamy tests, they were the product of bagged rachillae in non-natural conditions where the pollen was near the flowers after the inactive phase. As we ruled out anemophily, it appears that this species is entomophilous, which is consistent with the record of several insect groups visiting the inflorescences of other species [15], as well as most of the Arecaceae [12]. Entomophily appears to be a general trait in *Butia* palms. Their relationship with pollinators is essential for reproduction, maintenance of pollinator populations, and the production of fruits, which serve as an important food source for wildlife.

### 3.3. Self-Pollination

In Arecaceae, protandry is the general rule, and protogyny is the exception [16,34,35]. Protandry and dichogamy have been reported in four *Butia* species [23,36,37,38], and were confirmed for *B. lallemantii* in our study. These mechanisms are considered a way to prevent self-fertilization in Angiosperms [39,40]. Nevertheless, in *B. lallemantii,* geitonogamy is possible because the species is caespitose, and inflorescences of the same clump or ramet in different phenophases may overlap during their anthesis. Thus, the fruits formed by natural pollination may stem from allogamy or geitonogamy. Self-compatibility has been reported for two protandrous and solitary palms of the genus *Butia*: *B. capitata* [37] and *B. eriospatha* [23]. The case of *B. lallemantii* is the first known instance of a caespitose species in this genus exhibiting self-compatibility from both hand-made autogamy and geitonogamy. In plants, avoiding self-pollination creates intense selective pressure [41]. Self-compatibility increases the chances of fertilization and seed production in isolated populations where cross-pollination may be limited, but reduces the genetic variability and increases the probability of endogamy [42,43]. The anthesis of pistillate and staminate flowers in the same plant, but in different ramets, allows the occurrence of insect-mediated geitonogamy.

Autogamy tests were statistically different between them: spontaneous self-pollinated flowers formed 1% of fruits, while the manual procedure reached 77.8% success. The rachillae for the self-pollination test were isolated from the wind and insects. The pollination of the flowers in the test for spontaneous autogamy may be related to the transfer of the pollen deposited in the bag due to the balancing of the inflorescences by the wind. Instead, the hand-pollinated flowers received the pollen directly on the stigma, ensuring higher pollination success. For cross-pollinated tests, we obtained fewer differences: 83.3% for natural pollination and 84.4% for hand-pollinated allogamy. In this case, the effectiveness of the pollinators was high, but the hand-pollination was more effective.

It is important to note that all pollination treatments applied may potentially result in the production of apomictic seeds [44,45]. However, for *B. lallemantii*, apomixis was ruled out because the agamospermy test did not result in any fruit formation from the treated flowers. Apomixis is reported in less than 1% of Angiosperms, and it is present in more than 300 species [44,46]. It is an oddity in Arecaceae, reported for only a few species such as *Bactris gasipaes* Kunth var. *gasipaes* [47], *Mauritia flexuosa* L. f. [48], *Phoenix dactylifera* L., and *Chamaedorea radicalis* Mart. [49]. To date, none of the *Butia* species studied have been reported as apomictic.

### 3.4. Pollen and Nectar; Generalized Floral Rewards

In agreement with our hypothesis, we observed the primary pollinators of *B. lallemantii* feeding on flower pollen, nectar, or both while visiting inflorescences in the staminate and pistillate phases, consistent with reports from other species of *Butia* [15]. These appear to be the most common floral rewards in the genus [30,50], except for the caespitose *B. buenopolensis* Sant’Anna-Santos, whose staminodes produce oil-like droplets [38].

The production of pollen, nectar, or tissues consumed by the floral visitors, the large inflorescences, the diurnal anthesis, the exposed reproductive structures, and the potential for providing shelter, breeding, and oviposition sites offered by palms attract many animals, some of which may act as pollinators [12,13,51]. Consequently, pollination in Arecaeae is mainly mediated by insects, and the principal pollination syndromes are melittophily (Hymenoptera), cantharophily (Coleoptera), and myophily (Diptera), all associated with nectar and pollen [12,13,16]. The high presence of different species of insects in the inflorescence promotes many ecological relations and multitrophic interactions such as predation, food competition, and reproduction [52,53].

### 3.5. Native Pollinators Potentially at Risk

Based on our research, we can assert that the main pollinators of *B. lallemantii* are bees, with other groups of insects having less relevance. This is mainly due to the bees’ behavior, which promotes pollen transfer to the pistillate inflorescences, as reported for other *Butia* species. But we want to draw attention to the high relevance of the European honeybee in this process. It was registered in almost all the inflorescences competing with native bees for floral resources, with the advantage of their larger size and larger groups, a phenomenon reported in many countries around the world [54,55,56]. Some of the negative effects of *A. mellifera* on native bees are the reduction in fecundity [57], reduction in visitation rates by native insects [56], change in foraging behavior due to aggressive encounters [54,55], reduction in the quality of nutrition, and reorganization of the species interaction [58]. All these traits affect the pollination process and community stability and may have evolutionary implications [58,59].

All these facts above complicate the situation of the native bees, which are currently also facing problems related to habitat loss, the use of agrochemicals, climate change, and the introduction of pathogens [60,61]. In the case of the palm population we studied, local dwellers installed many honeybee hives to take advantage of the floral resources from the crops around them. Inevitably, honeybees search for resources in the native flora, and the abundance of inflorescences of *B*. *lallemantii* provides an opportunity. In consequence, native bee species competing with *A. mellifera* could be negatively impacted in their populations, foraging behavior, and ecological interactions [59,61,62]. The pollination of *B. lallemantii* may not appear to be impacted by the presence of *A. mellifera* because the production of fruits is over 80%. However, the habitat of the native bees is in constant decline, while honeybees continue to occupy more space in the agriculture and food industries. The potential detrimental effects of *A. mellifera* on the pollination of *B. lallemantii*, however, should be tested through rigorous experimental designs [63].

### 3.6. Conservation of the Dwarf Butiá

The Dwarf Butiá is at risk of extinction and is categorized as endangered (EN) for the Rio Grande do Sul state due to the reduction in populations and habitat degradation by human activities such as agriculture, livestock farming, and silviculture [64,65]. The few known populations grow on sandy and rocky soils in a small area spanning three Brazilian municipalities and part of the north of Uruguay. Furthermore, the population reported in the Paredão Private Natural Heritage Reserve is the only one in a protected area [24]. Therefore, it is necessary to establish protected areas in the municipalities where the populations occur to preserve and conserve the remaining populations and genetic diversity in situ, ensuring their ability to react to changing environmental conditions. [24,66,67].

The diverse possible products from *B. lallemantii* have the potential for agribusiness and sustainable uses, making it a suitable species for inclusion in diversified agroecosystems [68]. The production of fruit pulp and its derivates can improve productivity, diversify yields, and introduce new floral resources to the system, benefiting biodiversity maintenance [69,70,71]. Research initiatives are necessary for the domestication of this palm to recognize its value as a source of raw material for industry, determine its capacity for carbon sequestration, and establish its resilience limits, essential features to deal with climate change [72,73]. Cultural and economic knowledge about *B. lallemantii* is a valuable tool to promote government policies that ensure its conservation and sustainable use [30,73]. All available information about its ecology and ethnobotany helps to understand the importance of the *Butia* palm grooves and their integration into the identity of the region [25]. Public policies are needed to promote their sustainable use, conserve the grassy matrix, and protect the biodiversity associated with the *B. lallemantii* populations and their environment [25,74]. The Dwarf Butiá is socially, economically, and ecologically important in the context of the Pampa biome, an essential element of its identity that requires protection to ensure its conservation. This palm was used for a long time as a source of vegetable manure obtained from the leaves and used to stuff mattresses and upholstery. In the present, leaf fibers and endocarps are used for handicrafts, fruits for human food, juice, and jam [75].

Recent studies have demonstrated the potential uses for this palm in the restoration of degraded areas, biochemistry, and industry [27,29,76]. Experiments on the survival, growth, and development of *B. lallemantii* ramets with the use of different substrate compositions have been performed with promising results [26,28]. Also, the use of ramets in revegetating sandblasted soil shows a notable potential for ecological restoration in the Pampa [27]. Establishing a methodology to produce seedlings from seeds is crucial because *Butia* species are known for their difficulty in germinating [77,78]. In the case of *B. lallemantii,* it takes up to four years for the emergence of the eophyll (R. Singer, pers.com). The owner uses the study area for winter grazing; the cattle forage on the *B. lallemantii* leaves and other plants without apparent adverse effects (L. Oliveira, pers.com.).

However, studies are needed to establish the real impact of grazing on the flora and fauna growing with the Dwarf Butiá. Other uses reported for *Butia* species must be explored for *B. lallemantii* to expand its perspectives for sustainable use. For example, the roasted and ground endocarps of some *Butia* species, frequently discarded, are used to prepare a beverage like coffee called “café de coquito” in Uruguay [79]. In addition, artisans produce a paper sheet used for handicrafts from the pulp of the dried fruit of *B. capitata*, in a process analogous to recycled paper [80]. The amino acid content, fatty acid profiles, and phenolic compounds of *B. lallemantii* were recently reported [76], determining a well-balanced and large quantity of each amino acid and a fatty acid profile resembling this of *B. odorata* var. *pulposa* (Barb. Rodr.) Becc. In addition, the fruits present caffeic acid, a potential source for the food and medicine industries. Furthermore, the fibers obtained from the petiole were evaluated as reinforcement of polymeric composites, with positive results for their use in industry [29]. Finally, the landscape associated with *B. lallemantii* populations is of cultural, historical, and economic importance for the human population interacting with the “Butiazais” [81], which may be used to promote diverse forms of tourism and conservation in the region.

## 4. Materials and Methods

### 4.1. Study Area

We conducted the study in the Brazilian Pampa in a preserved area of approx. 50 ha. located in the Municipality of São Francisco de Assis, RS (29°32′59″ S, 55°7′51″ W; 180 m asl). The study area is surrounded by crops and pastures and harbors a *B. lallemantii* population with enough individuals for the experiments and observations. The site is in the South Temperate Zone with climate conditions characteristics of the Cfa group of the Köeppen classification: warm temperate, humid, with a hot summer, four seasons with regular precipitation rates, and rainfall that increases in autumn and winter [7,82,83]. The temperature varies from 10 to 30 °C, the annual precipitation is from 1600 to 1900 mm, and the relative humidity is around 75%. The experiments and observations were carried out in December 2021 and January 2022, at the beginning of the summer season.

### 4.2. Studied Species

**Figure 5:**
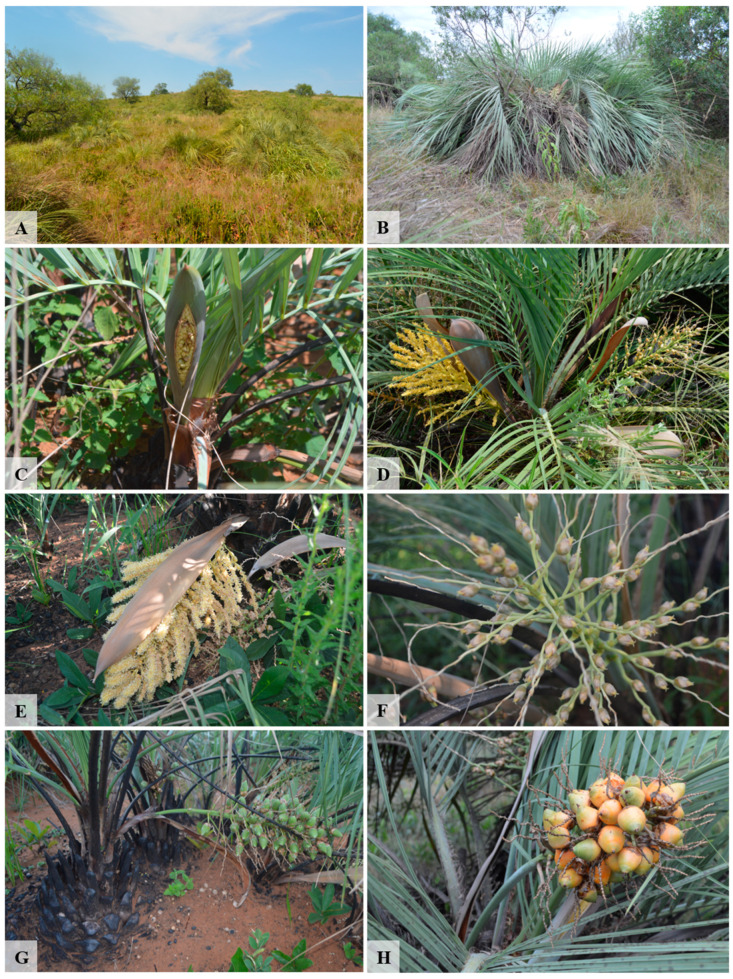
Habitat and reproductive structures of *Butia lallemantii* (Arecaceae). (**A**) Population studied; (**B**) Habit; (**C**) Opening spathe; (**D**) Palm with inflorescences in staminate and pistillate phases; (**E**) Staminate inflorescence; (**F**) Pistillate inflorescence; (**G**) unripe and (**H**) ripe infructescence.

*Butia lallemantii* is a cespitose palm tree, up to 1.7 m, with pinnate, glabrous, and arcuate leaves that form a hemispheric crown (Figure 5A,B). The inflorescences are erect, concealed by a greenish spathe up to 50 cm long (Figure 5C–F). The fruits are ovate-lanceolate up to 3.5 cm, yellow-orange when ripe, with edible pulp (Figure 5G,H) (detailed fruit morphology in [24,84,85]. The anthesis occurs in spring and summer, and the fructification occurs in summer and autumn [20]. This species occurs naturally in the Pampa of Brazil and Uruguay in rocky soils, sandy soils, and sandbanks. Currently, it is categorized as Endangered for the state of Rio Grande do Sul, Brazil, under the code A4cd: population reduction by causes that have not ceased and/or are not reversible [65,86].

### 4.3. Inflorescence, Anthesis, and Flower Duration

We sampled five plants, taking one near-to-open inflorescence from each, to measure morphological variables. The length, diameter at the base, and maximum diameter of the spathe were measured after the spathe was opened longitudinally with a knife to expose the inflorescence. The peduncle and rachis length and diameter measurements were also taken, along with the number of rachillae per inflorescence. In each inflorescence, we sampled three rachillae from the proximal, three from the middle, and four from the distal zone. We measured the diameter, total length, and length of staminate and pistillate zones and counted pistillate and staminate flowers. For the terminology related to sexual systems in Arecaceae, we follow Loo et al. [39].

**Figure 6:**
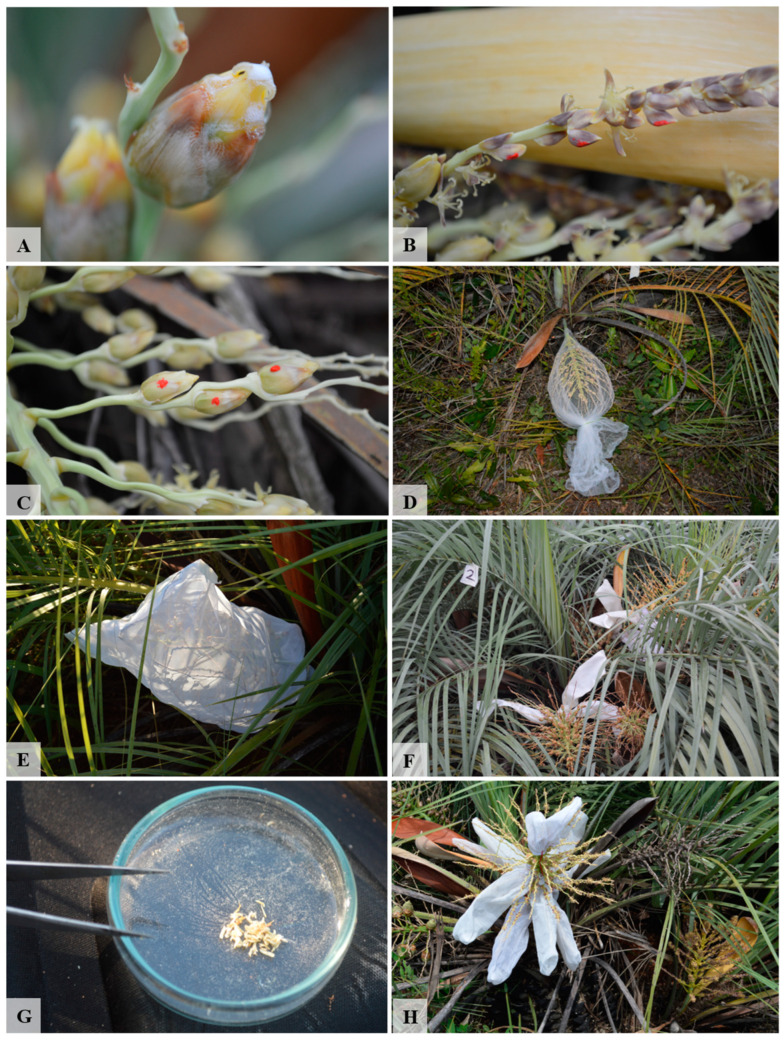
Breeding system experiments in *Butia lallemantii* in the Brazilian Pampa. (**A**) Peroxidase test in a pistillate flower; (**B**) Staminate flowers marked; (**C**) Pistillate flowers marked; (**D**) Inflorescence isolated with tulle fabric for wind-pollination test; (**E**) Inflorescence isolated with thin fabric for insect-pollination test; (**F**) Plant with treated inflorescences from different clumps; (**G**) Pollen extracted for the experiments; (**H**) Inflorescence with experiments developed in different rachillae.

To determine the length of the anthesis and its phenophases, we marked twenty individuals of *B. lallemantii* with inflorescences on the first day of anthesis, which was about to open. Then, the daily development of the inflorescence was monitored until the non-fertilized flowers fell. To test the receptivity of the pistillate flowers, we used the peroxidase test on five flowers per day and observed the reaction in the stigma (Figure 6A). The duration of pistillate and staminate flowers was recorded using five individuals in each phenophase by marking five unopened buds of each flower type per inflorescence and observing them hourly and daily, respectively, until the fall of each marked flower (Figure 6B,C). The inflorescences in the pistillate phase were isolated with tulle bags to avoid pollination.

### 4.4. Test for Pollinator-Dependency

To test for the dependence of pollination vectors on reproduction, we isolated 20 inflorescences of *B. lallemantii* before the anthesis, each one in a different individual. We designated ten inflorescences to test for wind pollination. These inflorescences were isolated from insects with a tulle bag that allows wind-borne pollen to reach the flowers and avoids the arrival and contact with animal floral visitors (Figure 6D) [87,88]. In addition, we used ten inflorescences to test for insect-dependent pollination. These inflorescences were isolated with thin fabric bags to exclude the arrival of wind-borne pollen and insects (Figure 6E). In both cases, fertilized pistillate flowers were searched for in each inflorescence after 15 days from the start of their anthesis.

### 4.5. Breeding System Experiments

**Figure 7:**
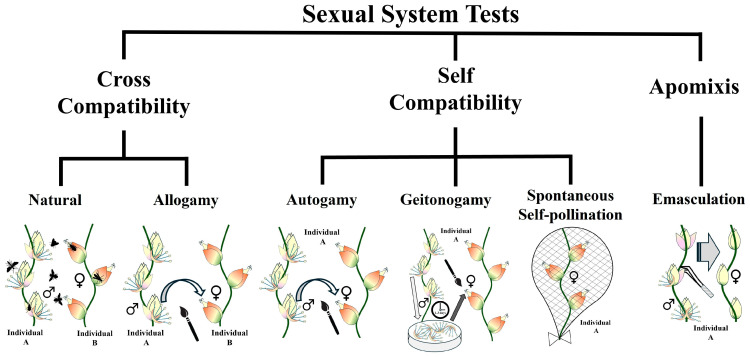
Breeding system experiments applied to *Butia lallemantii* in the Brazilian Pampa. Individuals A and B represent different plants used for the treatments.

The clonal growth is characteristic of *B. lallemantii*; it enables the simultaneous presence of inflorescences in the pistillate phase and others in the staminate, in the same ramet or on different ramets of the same individual (Figure 6F) [20]. Therefore, to understand the breeding system of this species, we performed the following experiments: tests for agamospermy, tests for cross-pollination, and tests for self-incompatibility (Figure 7). To test for agamospermy, we cut off the staminate flowers of the triads, isolated the rachillae with only pistillate flowers, and evaluated the formation of apomictic fruits [14,89]. To test for cross-pollination, we developed a xenogamy test for fertilizing pistillate flowers with pollen from a different individual [22,89]. Natural fruit formation was studied by leaving rachillae uncovered, thus allowing access to floral visitors [22,90].

Self-incompatibility/compatibility was tested through spontaneous self-pollination, hand-made self-pollination (thereafter, autogamy), and hand-made geitonogamy (thereafter, geitonogamy) (see below for details). For spontaneous self-pollination, we bagged the rachilla on the day of the spathe opening to observe if autonomous pollination occurred thereafter [22,91]. We tested for autogamy by fertilizing the pistillate flowers with pollen from the same inflorescence saved from the staminate phase [22,92]. For geitonogamy, pistillate flowers were fertilized with pollen from a different inflorescence on the same individual [14]. All the rachillae treated were isolated to avoid the arrival of pollen and the interaction with floral visitors, except for the rachilla in the natural pollination test. As a whole, we used ten individuals to test the breeding system. For each treatment, we used three rachillae and three flowers per rachilla in each individual, totaling 90 flowers per treatment.

We identified and marked palms with at least one inflorescence in the neutral phase (see Section 2) and one on the first day of the pistillate phase in either the same ramet or different ramets of the same individual. In the neutral phase of the inflorescence, we isolated three rachillae for hand-pollinated allogamy, and three for geitonogamy, applying the tests when the pistillate phase started. In the inflorescence on the first day of the staminate phase, we selected three rachillae, removed all the staminate flowers, and isolated them for the agamospermy test; we repeated the procedure for manual autogamy tests. Three rachillae were isolated with all the flowers to test for autogamy.

To obtain the pollen used in the geitonogamy test, we isolated one rachilla in the staminate phase the previous day to avoid floral visitors. Then, on the morning of the test day, we took staminate flowers opening or recently opened and deposited them in a Petri dish, cut off the stamens, and then smashed them with a tweezer to expose the pollen (Figure 6G). Finally, we used a brush to take the pollen and deposit it in the stigma of the treated flower. The same procedure was followed for the allogamy test, but using staminate flowers from another individual. We saved flowers from the last day of the staminate phase in a paper bag and waited for the pistillate phase of the same inflorescence to apply the autogamy test following the same procedure.

To compare the results of the treatments applied, we used an Analysis of Variance—ANOVA test in R language [93]. First, we performed Lillieforse normality tests for each group of data using the package nortest [94]. Then, we performed the analysis only with normally distributed data to detect significant differences between treatments. Finally, we used a post hoc test of Tukey to find which treatments were different. Additionally, we estimated the Index of Self-Incompatibility for *B. lallemantii* as the quotient of the number of fruits formed by self-pollination divided by the cross-pollination ones to evaluate the degree of genetic compatibility [95,96]. We also measured the reproductive efficiency, marking ten inflorescences exposed to natural pollination, counting the number of pistillate flowers in 3 rachillas, and following the number of fruits formed and pistillate flowers lost per inflorescence. Then, we calculate the reproductive efficiency as the ratio between fruits developed and pistillate flowers produced [97].

### 4.6. Floral Rewards and Floral Visitors

We observed the inflorescences of five individuals of *B. lallemantii* in the staminate and pistillate phases to check the consumption of pollen and nectar by the floral visitors. We used a Nikon D3200 (Melville, NY, USA) to take photos and videos to record the insect activity around the floral rewards. To identify the floral visitors of this species, we spent 30 h collecting them with an insect aspirator and 30 h observing and recording their behavior in both the male and female phases. Collections and observations were made in sessions of 20 min per plant, from 7 to 10 h in the morning, and from 15 to 18. We also developed 10 h of nocturnal observations in five sessions of two hours per night. The insect collections were preserved in plastic vials with alcohol and posteriorly deposited at the Museum of Natural Sciences of Rio Grande do Sul (Museu de Ciências Naturais da Secretaria do Meio Ambiente e Infraestrutura do Rio Grande do Sul—SEMA/RS). Identification of the floral visitors was made to the lowest possible taxonomic level, with help from specialists and comparison with specimens reported for other *Butia* species.

#### Pollinators

Pollination is the transfer of pollen to the conspecific stigma, which leads to fertilization and fruit production. It can be mediated by water or wind, but most wild and cultivated plants rely on animals for this process [60]. Thus, we define the pollinators of *B. lallemantii* as the floral visitors observed in both phenophases (male and female), exhibiting behavioral and morphological features that enable them to carry pollen from the anthers to the stigmatic surface. In addition, we determined the importance of each pollinator for the pollination process by analyzing the length of time spent by the pollinator during the flower visit as well as the frequency of their visits.

## 5. Conclusions

Complete breeding systems and pollination studies are needed to improve our knowledge about Arecaceae ecological relationships and implement suitable conservation and rational use strategies. Pollination in *B. lallemantii* is entomophilous, a trait that promotes cross-pollination and is likely shared by all the *Butia* species studied to date. This species is self-compatible and geitonogamous, a reproductive feature that allows reproduction between flowers of the same individual and ramets of the same clump, with ecological advantages and disadvantages. Pollen and nectar from the inflorescences of *B. lallemantii* are the key food resources for floral visitors and promote ecological interactions around the inflorescence. The primary pollinators of the Dwarf Palm were native bees of the subfamilies Meliponinae and Halictinae and the introduced *Apis mellifera* (Apinae), with less incidence of Curculionidae, Muscidae, and Chrysomelidae. *A. mellifera* may negatively impact native pollinators, especially native bees, reducing their populations, competing for food and space, and modifying their ecological interactions. *Butia lallemantii* has the potential to serve as a raw material source for various industries, making it a valuable candidate to diversify agroecosystems while also preserving its social, cultural, economic, and ecological significance in the Pampa biome.

## Figures and Tables

**Table 1:** Morphology of *Butia lallemantii* inflorescence structures (*n* = 5).

Structure	Mean (±SE)	Max–Min.
Inflorescence length	65.76 (±7.38) cm	45.2–84.2
Peduncle length	30.64 (±6.66) cm	19.4–41.5
Peduncle diameter	1.03 (±0.21) cm	0.8–1.3
Rachis length	30.22 (±3.62) cm	21.7–47.3
Rachis diameter	1.1 (±0.13) cm	0.7–1.4
Rachillae number	58.4 (±6.11)	48–64
Total flowers	80.36 (±9.99)	51–124
Total pistillate flowers	2.56 (±1.02)	0–6
Total staminate flowers	78.36 (±8.59)	51–122
Spatha length	65.67 (±6.21) cm	45.2–84.2
Base diameter	2.9 (±0.29) cm	2.3–3.9
Maximum diameter	8.38 (±0.95) cm	5.6–10.5

**Table 2:** Results of the breeding system experiments developed on *Butia lallemantii* flowers.

Treatment	Flowers Treated	Fruits Formed	Fruits Formed (%)
Allogamy	90	76	84.4
Natural pollination	90	75	83.3
Autogamy	90	70	77.8
Geitonogamy	90	63	70
Spont. Self-pollination	90	3	3.3
Apomixis	90	0	0

**Table 3:** Comparison of the breeding system experiments developed on *Butia lallemantii*. The results of the Tukey test were used to compare the results of the pollination treatments applied.

Treatment	Autogamy	Geitonogamy	Allogamy	Natural Pollination
Autogamy	1.00	--	--	--
Geitonogamy	0.478	1.00	--	--
Allogamy	0.641	0.018 *	1.00	--
Natural pollination	0.793	0.036 *	0.999	1.00

* Significant statistical differences (*p* < 0.001).

**Table 4:** Species per family of the floral visitors registered in *Butia lallemantii,* discriminated by species registered on staminate and pistillate inflorescences and in both phenophases.

Order/Family	Total	Staminate	Pistillate	Both	(%) Visitors	(%) Pollinators
**Diptera**	**9**	**3**	**8**	**1**	**14.8**	**3.7**
Drosophilidae	1	0	1	0	1.6	0.0
Phoridae	1	0	1	0	1.6	0.0
Muscidae	6	2	6	1	9.8	3.7
Syrphidae	1	1	0	0	1.6	0.0
**Coleoptera**	**16**	**9**	**13**	**6**	**26.2**	**22.2**
Cerambycidae	1	1	0	0	1.6	0.0
Chrysomelidae	3	2	2	1	4.9	3.7
Curculionidae	7	5	7	5	11.5	18.5
Elateridae	1	0	1	0	1.6	0.0
Lampyridae	1	1	0	0	1.6	0.0
Nitidulidae	2	0	2	0	3.3	0.0
Silvanidae	1	0	1	0	1.6	0.0
**Hemiptera**	**2**	**2**	**1**	**1**	**3.3**	**3.7**
Reduviidae *	2	2	1	1	3.3	3.7
**Hymenoptera**	**34**	**23**	**29**	**19**	**55.7**	**70.4**
Apidae	9	5	11	6	11.2	22.2
Halictidae	7	7	5	5	14.4	18.5
Formicidae	10	7	7	4	16.4	14.8
Vespidae	5	2	4	2	6.5	7.4
Tiphiidae	2	1	1	1	3.3	3.7
Andrenidae	1	1	1	1	1.6	3.7
**Total**	**61**	**37**	**51**	**27**	**100**	**100**

* The species was observed but not collected.

## Data Availability

Data are contained within the article and Supplementary Materials.

## Supplementary Materials

The following supporting information can be downloaded at: https://www.mdpi.com/article/10.3390/plants13111562/s1. Video S1: Main group of insect pollinators of *Butia lallemantii* Deble & Marchiori.
